# Microbial education plays a crucial role in harnessing the beneficial properties of microbiota for infectious disease protection in *Crassostrea gigas*

**DOI:** 10.1038/s41598-024-76096-4

**Published:** 2024-11-06

**Authors:** Luc Dantan, Prunelle Carcassonne, Lionel Degrémont, Benjamin Morga, Marie-Agnès Travers, Bruno Petton, Mickael Mege, Elise Maurouard, Jean-François Allienne, Gaëlle Courtay, Océane Romatif, Juliette Pouzadoux, Raphaël Lami, Laurent Intertaglia, Yannick Gueguen, Jeremie Vidal-Dupiol, Eve Toulza, Céline Cosseau

**Affiliations:** 1 IHPE, Univ Perpignan Via Domitia, CNRS, IFREMER, Univ Montpellier, Perpignan, France; 2 Ifremer, ASIM, La Tremblade, F- 17390 France; 3 IHPE, Univ Montpellier, CNRS, IFREMER, Univ Perpignan Via Domitia, Montpellier, France; 4 Univ Brest, CNRS, IRD, LEMAR, Ifremer, Plouzané, F-29280 France; 5 Laboratoire de Biodiversité et Biotechnologies Microbiennes, Sorbonne Université, CNRS, Observatoire Océanologique de Banyuls-sur-Mer, Avenue Pierre Fabre, Banyuls-sur- Mer, 66650 France; 6 Sorbonne Université, CNRS, Fédération de Recherche, Observatoire Océanologique de Banyuls-sur-Mer, Banyuls-sur-Mer, 66650 France; 7 MARBEC, Univ Montpellier, CNRS, Ifremer, IRD, Sète, France

**Keywords:** *Crassostrea gigas*, Microbial education, Oyster holobiont, OsHV-1 µVar, *Vibrio aestuarianus*, Immunology, Microbiology

## Abstract

The increase in marine diseases, particularly in economically important mollusks, is a growing concern. Among them, the Pacific oyster (*Crassostrea gigas*) production faces challenges from several diseases, such as the Pacific Oyster Mortality Syndrome (POMS) or vibriosis. The microbial education, which consists of exposing the host immune system to beneficial microorganisms during early life stages is a promising approach against diseases. This study explores the concept of microbial education using controlled and pathogen-free bacterial communities and assesses its protective effects against POMS and *Vibrio aestuarianus* infections, highlighting potential applications in oyster production. We demonstrate that it is possible to educate the oyster immune system by adding microorganisms during the larval stage. Adding culture based bacterial mixes to larvae protects only against the POMS disease while adding whole microbial communities from oyster donors protects against both POMS and vibriosis. The efficiency of immune protection depends both on oyster origin and on the composition of the bacterial mixes used for exposure. No preferential protection was observed when the oysters were stimulated with their sympatric strains. Furthermore, the added bacteria were not maintained into the oyster microbiota, but this bacterial addition induced long term changes in the microbiota composition and oyster immune gene expression. Our study reveals successful immune system education of oysters by introducing beneficial microorganisms during the larval stage. We improved the long-term resistance of oysters against critical diseases (POMS disease and *Vibrio aestuarianus* infections) highlighting the potential of microbial education in aquaculture.

**Supplementary Information:**

The online version contains supplementary material available at 10.1038/s41598-024-76096-4.

## Introduction

The Pacific oyster *Crassostrea gigas* (also known as *Magallana gigas*) stands as the most widely cultivated oyster species in the world, underpinning a substantial proportion of the aquaculture industry^1^. However, the production of *C. gigas* faces significant challenges due to recurring infectious diseases, inflicting high mortalities each year^2–5^. Two prevalent infections – the Pacific Oyster Mortality Syndrome (POMS) caused by the Ostreid herpesvirus type 1 µVariant (OsHV-1 µVar) and vibriosis initiated by *Vibrio aestuarianus* infection – are primarily responsible for these alarming mortalities. POMS is a complex and polymicrobial disease that preferentially affects younger oysters and can decimate up to 100% of spat on French farms^6,7^. The infection by OsHV-1 µVar marks a critical step in the progression of POMS, inducing an immunocompromised state in oysters by altering haemocytes physiology^6,8^. This leads to a dysbiosis of oyster microbiota and results in colonisation by opportunistic bacteria and death of the oyster^6,8,9^. On the other hand, *V. aestuarianus* is another harmful primary pathogen with chronic mortality reaching a cumulative mortality rate up to 30%. This loss induces important economic consequences since it preferentially infects market size oysters that have been raised for several years^10,11^.

Efforts to combat these infectious diseases have spawned various approaches based on the increasing knowledge and resources available on oysters. Genetic selection is a promising avenue which aims at selecting pathogen-resistant oysters^12,13^. However, this approach exhibits limitations such as the potential selection of trade-offs which could counter select traits important for the commercial value of *C. gigas*. Moreover, the demonstration of the existence of immune priming in *C. gigas* has opened up a whole new field of applications based on the use of viral mimics^14–17^. However, this innovative approach only protects against POMS infections^15,18^. A diversity of studies on oyster-microbiota interactions have also opened a new field of investigations consisting of identifying bacteria that are beneficial for their associated host during adverse conditions^9,19–21^. Research on disease prevention in molluscs based on the use of probiotics has been ongoing for decades but has yet to see widespread applications on farms^22–25^. While several pre/probiotic-based methods to mitigate infectious diseases have demonstrated success in shrimp hatcheries^26–28^, their application in oyster farming, particularly in open-sea environments, faces distinct challenges and limitations. Oysters, as filter-feeding organisms, often face complex microbial interactions in their natural habitats^29^. Consequently, achieving and maintaining a precise balance of beneficial microorganisms through the addition of probiotics can be challenging. Additionally, their culture in open-sea present limitations in the implementation of probiotics.

The concept of microbial education, consists of exposing the host immune system to beneficial microorganisms during early development^30,31^. This is because early life stages represent critical periods of growth and development during which the host’s immune system is still maturing^32^. This strategy offers significant advantages for oysters, as it can confer a protective effect while allowing exposure in hatcheries during the larval phase in controlled environments^33^. Numerous studies have shown that a proper host-microbiota interaction during the early development play an important role in the long-term host immune responses in a wide range of marine organisms^34–37^. In this context, Fallet and colleagues^20^ explored the potential of using wild-microbiota to educate the immune system of *C. gigas*. Through a ten-day exposure of *C. gigas* larvae to a whole microbiota from donor oysters, long-term beneficial effects were induced. The microbiota-exposed oysters exhibited enhanced resistance to OsHV-1 µVar, resulting in improved survival rates compared to non-exposed counterparts. This study underscored the crucial role of the microbiota in oyster immune system education, suggesting potential applications in commercial hatcheries. However, concerns regarding exposure to hazardous uncontrolled microbial communities transferred from donor oysters necessitate a cautious approach as it might contain primary or opportunistic pathogens. Indeed, prior to the recipient larvae exposure performed in Fallet et al. study, the donor oysters were placed in farming area during a non-infectious period to allow oysters to capture the maximum diversity of field microorganisms. Then, these donor oysters were placed in the rearing tanks during larval development where they transmitted their highly diverse microbial community to the recipient larvae. Although the donor oysters were considered healthy^38,39^, the presence of undetectable pathogens cannot entirely be excluded. Furthermore, the composition of the microorganisms added to the tanks during ME exposure is random, which means that Fallet et al. study may have missed a specific response that could be expected when adding a particular bacterial strain or mix. Here, our study aimed to explore the feasibility of microbial education in oyster larvae while considering and mitigating the risks associated with uncontrolled transfer of hazardous microorganisms found in wild microbiota. We investigated whether exposing oyster larvae to a controlled, pathogen-free bacterial community from donor oysters that had always been maintained in biosecured facilities could confer protective effects against POMS and *V. aestuarianus* infection. Additionally, we examined the feasibility of microbial education using a reduced synthetic bacterial community composed of cultivable bacteria isolated from disease resistant oysters. For this purpose, we developed and tested multi-strain bacterial mixes originating from the same geographical areas as the recipient oyster populations used in this study. Our comprehensive assays encompassed three distinct oyster populations from the Atlantic Ocean (Brest Bay, La Tremblade in Marennes-Oleron Bay, and Arcachon Bay) and one from the Mediterranean Sea (Thau Lagoon), enabling an in-depth exploration of the potential differential effects of bacterial exposure to either sympatric or allopatric oyster populations.

## Materials and methods

### Oyster sampling

Oysters were collected along the French Atlantic coast, during two different sampling campaigns (in February 2020 and November 2020), while it was only in November 2020 for the Mediterranean site due to COVID-19 restrictions arisen earlier in the year. For the Atlantic coast, 3 sites were selected: the Brest Bay (Brittany, France; lat 48.3349572; long − 4.3189134), La Tremblade in Marennes-Oleron Bay (Nouvelle-Aquitaine, France; lat 45.8029675; long − 1.1534223) and the Arcachon Bay (Nouvelle-Aquitaine, France; lat 44.6813750; long − 1.1402178). For the Mediterranean coast, the selected site was the Thau Lagoon (Occitanie, France; lat 43.39404; long 3.58092). For each site, 5 oysters (average weight = 2.5 g) were randomly sampled. Hence, the sampled oysters were located on sites with a high density of oysters (wild and farmed) and have therefore survived an annual infectious episode of POMS, allowing us to assume that they were resistant to the disease but also in the window of permissiveness for *Vibrio aestuarianus* infection^40^. Based on these facts, we hypothesised that sampling bacteria from these disease-resistant oysters increases the likelihood of isolating beneficial bacteria.

### Isolation of cultivable bacteria from *Crassostrea gigas*

The five disease-resistant oysters sampled at each site were carefully brushed and washed to remove the sediments, epiphytes and epibionts present on the shell. The flesh of the animals was then individually crushed with an Ultra-Turrax T25 mixer (5 × 5 s) in 15 ml falcon tubes. The homogenised tissues were then diluted at 1:10, 1:100 and 1:1000 in sterile artificial seawater. One hundred microliters of each dilution was spread on two Marine Agar (MA) (Marine Agar Difco 2216) plates and incubated at 15–20 °C.

After a minimum incubation period of 3 days, bacterial colonies were selected according to their morphotypes. A maximum number of different morphotypes were selected to maximise the biodiversity in our sampling and were isolated by streaking a colony on a new MA plate and purified by two successive subcultures. Then, the pure cultures of individual bacteria were transferred onto Marine Broth (MB) tubes (Marine Broth Difco 2216) at 15–20 °C under constant agitation. After 48 h of growth, 500 µL of these cultures was used for cryopreservation in 35% glycerol (v/v) and placed in a -80 °C freezer. About 1 ml of the liquid culture was pelleted for further DNA extraction.

## DNA extraction and identification of the cultivable bacteria

DNA extraction of the bacterial strains isolated from oysters and cultivated on agar plates was carried out with the Wizard^®^ Genomic DNA Purification Kit (Promega) according to the manufacturer’s instructions. 16 S rRNA gene sequencing was performed on these samples to identify each bacterium from the collection. PCR and 16 S rRNA gene sequencing were performed by the Genoscreen sequencing facilities (http://www.genoscreen.fr/fr/). Briefly, two pairs of primers P8/PC535 (P8 5’-AGAGTTTGATCCTGGCTCAG; PC535 5’- GTATTACCGCGGCTGCTGGCAC) and 338 F/1040R (338 F 5’-CTCCTACGGGAGGCAG; 1040R 5’-GACACGAGCTGACGACA) were used for the PCR to amplify V1-V3 and V3-V5 region of the 16 S rRNA gene. PCR products were then purified with Sephadex-G50 gel (GE Healthcare) before analysis into ABI 3730XL capillary sequencer. The resulting sequences were then assembled by using DNA baser sequence assembly software (v4) (Heracle BioSoft, www.DnaBaser.com) and then added to the Ezbiocloud database^41^ in order to identify the taxonomy of the isolated bacteria composing the collection.

## Larval cytotoxic effect

Two day-old larvae (D stage) were distributed in the wells of a 6-well plate filled with three ml of sterile seawater at a density of 10 larvae/ml and maintained at a temperature of 20 °C and a 12:12 day: night photoperiod. Treatment (bacterial challenge with a single bacterial strain) and control (only sterile seawater) were each conducted in duplicate. The bacteria were cultivated from glycerol stock in 10 ml of Marine Broth (MB) for 24 h at 20 °C, and then, 1 ml of each bacterial culture was inoculated into 10 ml of fresh MB media and incubated at 20 °C under constant agitation. After 48 h of incubation, the OD_600_ was measured, and the appropriate amount of bacteria was collected before centrifugation at 4000 rpm for 2 min and the supernatant was discarded. The pellets were then resuspended in 10 ml of sterile seawater. Larvae were challenged by the addition of a target concentration of 10^7^ CFU/ml of each bacterial strain (Multiplicity of infection = 10^6^ bacteria per larvae). Larval mortality was recorded 48 h after the addition of bacteria by evaluation of active swimming and/or gut and cilia movement under a binocular microscope.

## Preparation of multi-strain bacterial mixes for interaction with oysters

Five multi-strain bacterial mixes were tested (Table 1): four site-specific multi-strain bacterial mixes composed of bacteria isolated from oysters sampled at each geographical site (Brest mix, La Tremblade mix, Arcachon mix and Thau mix) and a multi-site bacterial composed of bacteria isolated from oysters sampled on all the different sites. The bacteria were cultivated from glycerol stock in 10 ml of Marine Broth (MB) for 24 h at 20 °C, and then, 1 ml of each bacterial culture was inoculated into 50 ml of fresh MB media and incubated at 20 °C under constant agitation. After 48 h of incubation, the OD_600_ was measured, and a quantity of 3.10^8^ CFU was collected and pooled into a same mix for each cultivated bacterium. The mixes were then centrifuged at 4000 rpm for 2 min and the supernatant was discarded. The pellets were then resuspended in 10 ml of sterile seawater and added immediately to 30 L larval rearing tanks to a final concentration of 10^4^ CFU/ml for each bacterium.

## Oyster reproduction

A total of 150 wild oysters were randomly sampled from each geographic site as described above (Brest Bay, La Tremblade in Marennes-Oleron Bay, Arcachon Bay, Thau Lagoon) in order to generate 4 oyster populations (Brest, La Tremblade, Arcachon and Thau populations) accordingly to commercial oyster hatchery practices. Briefly, oyster genitors were transferred into the Ifremer hatchery facility in La Tremblade. To avoid eventual horizontal transmission of pathogens among populations, each was placed in separate tanks of 250 L in a flow through system with a water circulation rate of 500 L/h. Seawater temperature was gradually increased from 10 to 20 °C within one week and maintain to 20 °C to favour the gametogenesis. Broodstock were fed *ad libitum* with a mixture of phytoplankton (*Isochrysis galbana*,* Tetraselmis suecica*, and *Skeletonema costatum*). After 2 months, oysters were shucked and sexed by microscopic observation. Only fully mature oysters were used, representing between 20 and 23 genitors per population **(Supplementary File 1**,** Table ****S1****)**. Spermatozoa and oocytes were collected by stripping the gonad. For each population, sperm was collected individually for each male, while oocytes of all females were pooled. Eggs were sieved on a 20 μm and 100 μm screens to remove small and large debris, respectively, the eggs being retained on the 20 μm screen. Then, the pool of eggs was divided by the number of males, and each subgroup was fertilised by a male. Fifteen minutes after fertilisation, all subgroups were mixed, and all fertilised and unfertilised eggs were placed in fourteen 30 L tanks at a density of 34 to 100 eggs/mL (**Supplementary File 1**,** Table ****S2**). Thus, depending on the population, between one to three million eggs were added to each 30 L conical tank. Tanks were in a batch system containing 26 °C filtered and UV-treated seawater, supplemented with gentle air-bubbling. The larval farming density was 10 larvae/ml at day 2, and 3 larvae/ml at day 7. The seawater was changed three times per week, and the larvae were fed daily with *Isochrysis galbana*, supplemented with *Skeletonema costatum* from day 7. The physico-chemical parameters of the water and the microalgae ration used before distribution to the tanks were recorded every day during larval rearing steps (**Supplementary File 2: EDM Parameters**).

### Exposure of oyster larvae with microorganisms

**Fig. 1:**
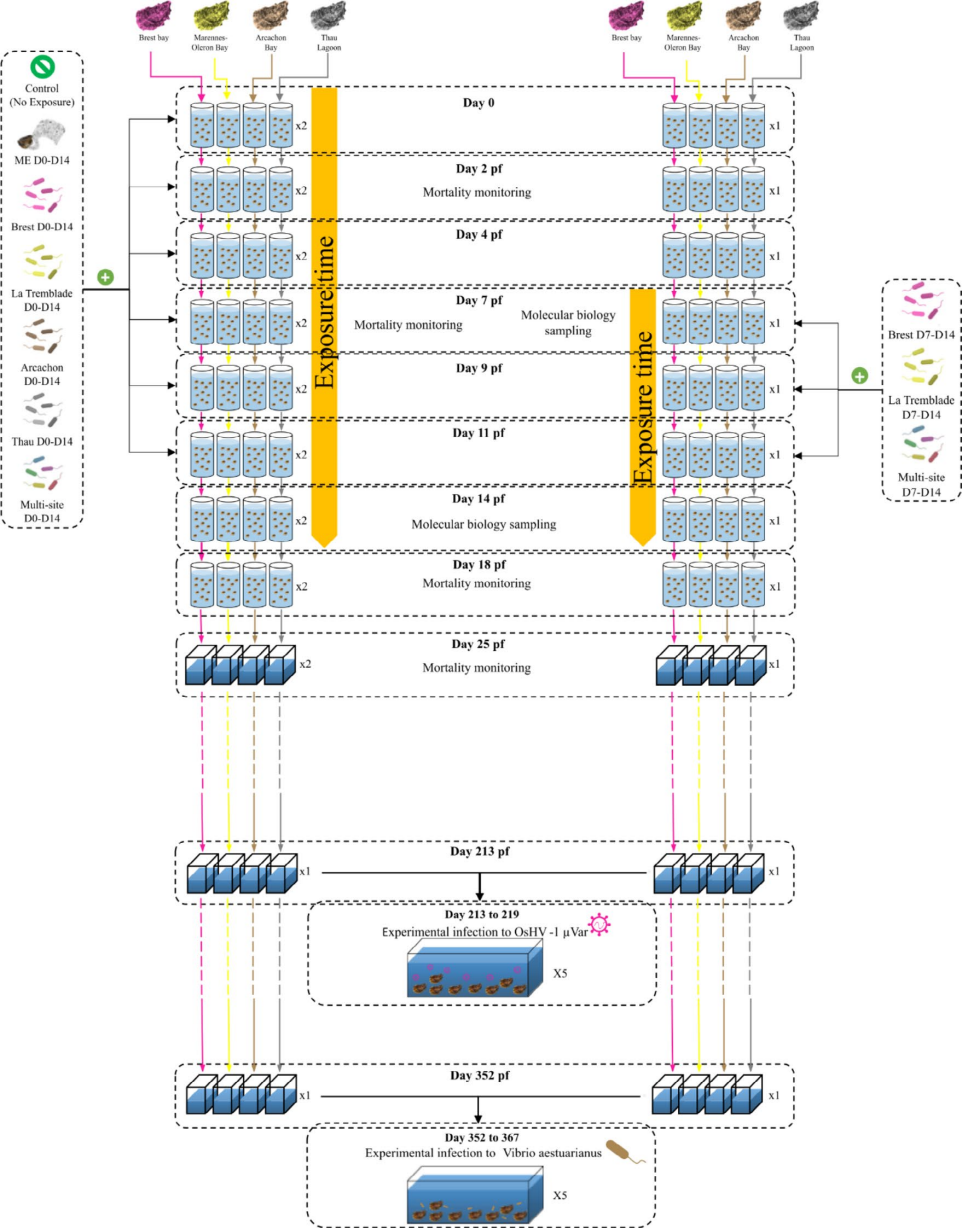
Overall experimental design for larval microbial exposure and experimental infections. Multi-parental reproduction was performed for the four oyster populations and the larvae were placed in 30 L tanks in a batch system containing 26 °C filtered and UV-treated seawater, supplemented with gentle air-bubbling. Three hours post-fertilisation (pf), larvae remained unexposed (2 tanks) or were exposed in duplicate to microbiota from donor oysters (ME seawater D0-D14) or to the five different multi-strains bacterial mixes (Brest D0-D14, La Tremblade D0-D14, Arcachon D0-D14, Thau D0-D14 and Multi-site D0-D14). This microorganism exposure was renewed three times per week and lasted for 14 days. In parallel, exposure to three multi-strains bacterial mixes (Brest D7-D14, La Tremblade D7-D14 and Multi-site D7-D14) was performed on older larvae between D7 and D14 pf. During the larval stage, seawater and larvae were sampled at days 2, 7, 11, 14, 18 and 25 pf to perform growth and mortality monitoring, or to perform molecular analysis. After the larval stage, spat grew in our controlled facility. At day 213 pf (approximately seven months old), a first set was used to carry out an experimental infection to OsHV-1 µVar, and on day 352 pf (approximately one year), a second set was used to perform a *V. Aestuarianus* experimental infection.

For each population, seven conditions were tested, each using two 30 L replicate tanks. Larvae were either unexposed or exposed to microbiota from donor oysters (ME seawater D0-D14) or to the five different multi-strains bacterial mixes at two different larval developmental windows (Brest D0-D14, Brest D7-D14, La Tremblade D0-D14, La Tremblade D7-D14, Arcachon D0-D14, Thau D0-D14 and Multi-site D0-D14, Multi-site D7-D14 (Fig. 1). For ME seawater D0-D14, larvae were exposed to the whole natural microbiota coming from healthy donor oysters (Microorganism-Enriched seawater = ME seawater). This microorganism community was introduced thanks to donor oysters of microbiota, which were placed into the rearing tank. Oyster donors of microbiota were NSI (Naissains Standardisés Ifremer, standardised Ifremer spats)^42,43^, which were always kept in controlled facilities using UV-treated seawater, strict biosecurity zoning and management procedures. The oysters were tested negative for the three main pathogens (*Vibrio coralliilyticus*, OsHV-1 µVar and *Haplosporidium costale*) of *C. gigas* from larvae to juveniles^10,44^. The microorganisms were added to the larvae either 3 h post-fertilization (pf) and at each water change until day 14 pf or from day 7 pf to day 14 pf (Fig. 1). The water changes at day 14 was performed without addition of the bacterial mixes. In this sense, the microbial exposure ended on day 14.

Larval survival was determined by counting the larvae on days 2, 7 and 18 for oysters exposed from day 0 pf to day 14 pf or on day 18 for oysters exposed from day 7 pf to day 14 pf. Fixation rate (i.e. rate of surviving larvae that have settled and metamorphosed to the spat stage) was determined at day 25 pf for all conditions. Larvae (pools of 10,000–20,000 individuals) were sampled either at day 7 pf or at day 14 pf, flash frozen in liquid nitrogen and stored at -80 °C for subsequent molecular analysis. After the rearing steps, only one replicate was kept to perform the experimental infections.

All oyster populations were kept in controlled facilities of the La Tremblade hatchery using UV-treated seawater until experimental infections by OsHV-1 µVar or *V. aestuarianus*.

## OsHV-1 µVar experimental infection by cohabitation

OsHV-1 µVar experimental infection was performed either on control or microorganisms exposed oysters population (seven-month-old, mean individual weight = 2.80 ± 0.69 g), except for the Thau population for which a part of the oysters were lost. Indeed, the high larval mortality of Thau oysters exposed to Brest D0-D14, La Tremblade D0-D14 and multi-site D0-D14 led to a complete loss of this oyster population. Furthermore, two other conditions were lost later due to technical problems at the hatchery (Thau oysters exposed to ME-seawater D0-D14 and Brest mix D7-D14). A randomised complete block design composed of five 50 L tanks (replicates) filled with filtered and UV-treated seawater and maintained at 20 °C with adequate aeration and no food supply was used. Each tank contained 12 oysters from each population exposed to each condition (total: 420 oysters per tank) (**Supplementary File 3**,** Figure ****S1**). A cohabitation protocol, adapted from^45^ was used as described. This approach starts with the injection of 100 µL of OsHV-1 µVar suspension (10^5^ OsHV-1 µVar genomic units) into the adductor muscle of pathogen-free oysters donors. This protocol allows for pathogen transmission through the natural infectious route to oysters of interest (recipient oysters). The OsHV-1 µVar donor oyster pool was composed of 25% of F15 family oysters, 25% of F14 family oysters which are POMS susceptible oysters^8^ and 50% of genetically diversified NSI oysters (~ 50% of susceptibility). The ratio was 1 donor oyster for 1 recipient oyster. Immediately after OsHV-1 µVar injection into donors (adductor muscle), recipient and donor oysters were uniformly distributed in each of the five experimental tanks. After 48 h of cohabitation, all donor oysters were removed from the tanks.

In each tank, one oyster from each population exposed to each condition was sampled just before the beginning of the experimental infection (t = 0 h infection) and three hours post cohabitation with OsHV-1 µVar donor oysters (t = 3 h infection) to perform molecular analysis on whole tissue samples. The shell was removed, and the whole flesh was flash frozen in liquid nitrogen and stored at -80 °C until it was grounded in liquid nitrogen (Retsch MM400 mill) to a powder that was then stored at -80 °C until DNA and RNA extraction.

The mortality was recorded daily during eight days. Dead recipient oysters were removed daily from the tanks.

During the mortality monitoring, 1 mL of water from each tank was sampled every day for the detection and quantification of OsHV-1 µVar.

### *Vibrio aestuarianus* experimental infection by cohabitation

*Vibrio aestuarianus* experimental infection was performed either on control or microorganisms exposed oysters (12 months old ; mean individual weight = 9.42 ± 1.29 g) with a cohabitation protocol previously described in^10^. A randomised complete block design composed of five 100 L replicate tanks filled with filtered and UV-treated seawater and maintained at 20 °C with adequate aeration and without food was used. Each tank contained 10 oysters from each population exposed to each condition (total: 350 oysters per tank) except for the Thau population for which a part of the oysters was lost as explained above. The *V. aestuarianus* 02/041 strain^46^ was grown in Zobell media at 22 °C for 24 h under agitation. The bacterial concentration was determined by spectrophotometry at 600 nm and adjusted to an optical density (OD_600_) of 1 representing 5.10^8^ bacteria/mL. *V. aestuarianus* donor oysters were injected into the adductor muscle with 100µL of the *V. aestuarianus* 02/041 suspension and were then equally distributed among the five tanks. The *V. aestuarianus* donor oyster population was composed of an equi-number of the four oyster populations produced for this project (Brest, La Tremblade, Arcachon and Thau populations). Immediately after *V. aestuarianus* injection into donors, donor oysters were added to the five tanks containing the recipient oysters. A ratio of 1 *V. aestuarianus* donor oyster for 1.5 recipient oyster was used. After 48 h of cohabitation, *V. aestuarianus* donor oysters were removed from the tanks.

The mortality was recorded daily during 15 days, and all the dead oysters were removed from the tanks. During the mortality monitoring, 1 mL of water from each tank was sampled every day for the detection and quantification of *V. aestuarianus*.

## Statistical analysis of oyster mortality

Oyster mortality rates were compared between the different microorganism exposure sets using survival analysis performed in R (v 4.2.1)^47^ with the package survminer (v 0.4.9) (https://cran.r-project.org/web/packages/survminer/index.html). The Kaplan-Meier method was used to represent the cumulative survival rate, and the log-rank test was used to determine the differences between conditions. A multivariate Cox proportional hazards regression model was used to compute Hazard-Ratio (HR) with a confidence interval of 95%.

### Genomic DNA extraction and sequencing of oysters and water

DNA was extracted from larvae (a pool of 10,000 to 20,000 individuals) collected during microorganisms exposure was extracted with the DNA from the tissue Macherey-Nagel kit according to the manufacturer’s protocol. Prior to 90 min of proteinase K lysis, an additional mechanical lysis was performed by vortexing samples with zirconia/silica beads (BioSpec). DNA from individual juvenile oyster tissues collected just before and during experimental infection was extracted from oyster powder with the DNA from tissue Macherey-Nagel kit according to the manufacturer’s protocol. Prior to 90 min of proteinase K lysis, an additional 12-min mechanical lysis (Retsch MM400 mill) was performed with zirconia/silica beads (BioSpec). DNA was extracted from water collected during microorganisms exposure and experimental infections was extracted with the DNA from tissue Macherey-Nagel tissue kit following the manufacturer’s support protocol for genomic DNA and viral DNA from blood samples.

DNA concentration and purity were checked with a Nanodrop ND-1000 spectrometer (Thermo Scientific).

### qPCR analysis

The detection and quantification of OsHV-1 µVar and *V. aestuarianus* was performed by real-time quantitative PCR. All amplification reactions were performed on Roche LightCycler^®^ 480 Real-Time thermocycler. Each reaction was carried out in triplicate in a total volume of 10 µL containing the DNA sample (2.5 µL), 5 µL of Takyon™ SYBER MasterMix blue dTTP (Eurogentec, ref UF-NSMT-B0701) and 1 µL at 500 nM of each primers for OsHV-1 µVar (OsHVDPFor5’-ATTGATGATGTGGATAATCTGTG and OsHVDPFor 5’-GGTAAATACCATTGGTCTTGTTCC)^48^ and for *V. aestuarianus* (DNAj-F 5′-GTATGAAATTTTAACTGACCCACAA and DNAj-R 5′-CAATTTCTTTCGAACAACCAC)^49^. The qPCR cycling conditions were as follows: 3 min at 95 °C, followed by 45 cycles of amplification at 95 °C for 10 s, 60 °C for 20 s, and 72 °C for 30 s. After these PCR cycles a melting temperature curve of the amplicon was generated to verify the specificity of the amplification. The DNA polymerase catalytic subunit amplification product cloned into the pCR4-TOPO vector was used as a standard at 10-fold dilutions ranging from 10^3^ to 10^10^ copies/ml for OsHV-1 µVar quantification and genomic DNA from *V. aestuarianus* ranging from 10^2^ to 10^7^ copies/ml for *V. aestuarianus* quantification. Absolute quantification of OsHV-1 µVar or *V. aestuarianus* was calculated by comparing the observed Cp values to the standard curve.

### 16 S rDNA library construction and sequencing

Library construction (with primers 341F 5’-CCTAYGGGRBGCASCAG and 806R 5’-GGACTACNNGGGTATCTAAT targeting the V3-V4 region of the 16 S rRNA gene)^50,51^ and sequencing on a MiSeq v2 (2 × 250 bp) were performed by ADNid (Montpellier, France).

### RNA extraction and sequencing

RNA was extracted from oyster powder (individual) by using the Direct-Zol RNA Miniprep Kit (Zymo Research) according to the manufacturer’s protocol. RNA concentration and purity were checked using a Nanodrop DN-1000 spectrometer (Thermo Scientific), and RNA integrity was analysed by capillary electrophoresis on a BioAnalyzer 2100 (Agilent).

### RNA-seq library construction and sequencing

RNA-Seq experiments were performed on 3 single individuals per condition. RNA-Seq library construction and sequencing were performed by the Bio-Environment Platform (University of Perpignan, France). Stranded libraries were constructed from 500 ng of total RNA using NEBNext UltraII and sequenced on a NextSeq550 instrument (Illumina) in single-end reads of 75 bp. The number of reads generated for each samples can be found in **Supplementary File 4**.

### Bioinformatic pipelines for 16 S rRNA gene barcoding analysis

Previously published barcoding datasets^8,19,20,52,53^ from 687 POMS-resistant and 664 POMS-sensitive oysters were re-analysed in this study in order to predict bacteria which were potentially associated with oyster POMS resistant phenotypes. Datasets used for these analyses are shown in **Supplementary File 1**,** Table ****S3**. These datasets were individually analysed under the Toulouse Galaxy instance (https://vm-galaxy-prod.toulouse.inra.fr/)^54^ with the Find Rapidly OTU with Galaxy Solution (FROGS) pipeline^55^. In brief, paired reads were merged using FLASH^56^. After denoising and primer/ adapter removal with cutadapt^57^, clustering was performed using SWARM^58^, which uses a novel clustering algorithm with a threshold (distance = 3) corresponding to the maximum number of differences between two OTUs. Chimeras were removed using VSEARCH^59^. We filtered out the dataset for singletons and performed an affiliation using BLAST against the Silva 16 S rDNA database (release 132) to produce an operational taxonomic unit (OTU) and affiliation tables. In order to identify bacterial taxa that were significantly overrepresented in the microbial community associated with POMS-resistant compared with POMS-sensitive oysters, the “LDA Effect Size” (LEfSe) method^60^ was used with a normalised relative abundance matrix. This method uses a Kruskal-Wallis test followed by a Wilcoxon test (pval ≤ 0.05) and then performs a linear discriminant analysis (LDA) and evaluate the effect size. The taxa with a LDA score greater than 2 were considered to be significantly enriched in the POMS-resistant compared to POMS-sensitive oysters.

Sequencing data obtained from the samples from this study were processed with the SAMBA (v 3.0.2) workflow developed by the SeBiMER (Ifremer’s Bioinformatics Core Facility). Briefly, Amplicon Sequence Variants (ASV) were constructed with DADA2^61^ and the QIIME2 dbOTU3 (v 2020.2) tools^62^, then, contaminations were removed with MicroDecon (v 1.0.2)^63^. Taxonomic assignment of ASVs was performed using a Bayesian classifier trained with the Silva database v.138 using the QIIME feature classifier^64^. Finally, community analysis and statistical analysis were performed in R (version 4.2.1)^47^ using the phyloseq (v 1.40.0)^65^ and Vegan (v 2.6-4)^66^ packages. Unique and overlapping ASVs of each sample group were plotted using the UpsetR package (v 1.4.0)^67^. For beta-diversity, the ASVs counts were preliminarily normalised with the “rarefy_even_depth” function (rngseed = 711) from the package phyloseq (v 1.40.0)^65^. Differences between groups were assessed by statistical analyses (Permutational Multivariate Analysis of Variance) using the adonis2 function implemented in vegan^66^.

In order to follow the long-term installation (or not) of each of the bacteria used in the multi-strain bacterial mixes in the oyster microbiota, 16 S rRNA gene sequences obtained during the identification of each of the bacteria composing the multi-strain bacterial mixes were used as a query for a similarity BLASTn search against all the ASV sequences from the dataset^68^. A mock community composed of equal amounts of DNA from the bacteria composing the multi-strain bacterial mixes were also used as a positive control to validate our search method. ASV sequences with a percentage of identity higher than 99% were considered to be present in the tested samples.

### Bioinformatic pipeline for RNA-Seq analysis

All data treatments were carried out under a local galaxy instance (http://bioinfo.univ-perp.fr)^54^. Reads quality was checked with FastQC (Babraham Bioinformatics) with default parameters (Galaxy Version 0.72). Adapters were removed using Trim Galore (Galaxy Version 0.6.3) (Babraham Bioinformatics). Reads were mapped to the *C. gigas* genome (assembly cgigas_uk_roslin_v1) using RNA STAR (Galaxy Version 2.7.8a) (**Supplementary File 4: RNAseq Mapping results)** and HTSeq-count^69^ was used to count the number of reads overlapping annotated genes (mode Union) (Galaxy Version 0.9.1). The differential gene expression levels were analysed with the DESeq2 R package (v 1.36.0)^70^. Finally, Rank-based Gene Ontology Analysis (GO_MWU package) was performed using adaptive clustering and a rank-based statistical test (Mann–Whitney U-test combined with adaptive clustering) with the following parameters: largest = 0.5; smallest = 10; clusterCutHeight = 0.25. The signed “-Log(adj pval)” (obtained from the DESeq2 analysis) was used as an input for the GO_MWU analysis. The R and Perl scripts used can be downloaded [https://github.com/z0on/GO_MWU]^71^.

## Results

### Twenty-three bacterial strains with potential beneficial effects were selected to generate the multi-strain bacterial mixes

**Fig. 2:**
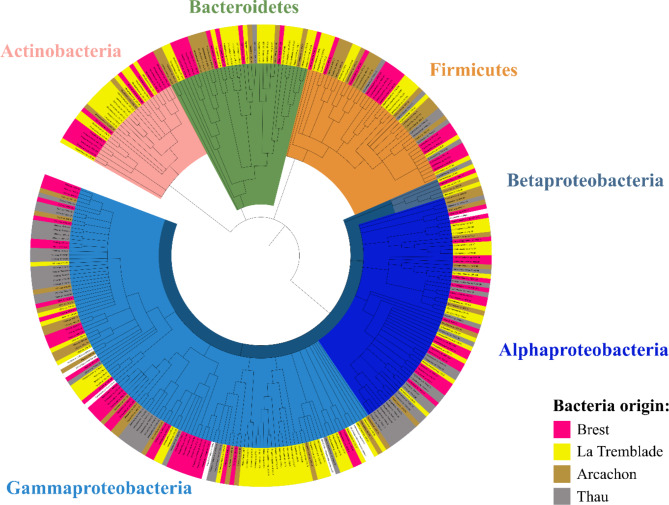
293 strains were identified in the bacterial collection sampled from POMS-resistant oysters. Phylogenetic tree of the 293 identified bacteria composing the collection of bacteria isolated from POMS-resistant oysters sampled in the Brest Bay (pink), La Tremblade in Marennes-Oleron Bay (yellow), the Arcachon Bay (brown) and the Thau Lagoon (grey) based on the V1-V5 loop alignment of bacterial 16 S rDNA by a Maximum likelihood method with the Tamura-Nei parameter model in MEGA X (301 sequences) and 1000 bootstrap replicates. The collection is composed by 62.8% of Proteobacteria (different shades of blue), 15.3% of Firmicutes (orange), 12.3% of Bacteroidetes (green) and 9.6% of Actinobacteria (salmon).

To isolate bacteria with potential beneficial effects against oyster infectious disease, we hypothesised that bacteria should be isolated from disease resistant oysters. For this purpose, wild oysters aged between 12 and 18 months were sampled close to farming areas. Oysters located in these areas are submitted to high pathogen pressure and have been shown to be resistant to POMS disease^72^. To maximise the biodiversity of the bacterial collection, oysters were sampled from 4 geographical French sites at two different seasons. A total of 334 bacteria were isolated (**Supplementary File 1**,** Table ****S4**); from which 166 bacteria were obtained from the February 2020 sampling campaign, and 168 bacteria were obtained from the November 2020 sampling campaign. This corresponded to 97, 144, 56, and 67 bacteria isolated from Brest, La Tremblade, Arcachon, and Thau sites, respectively. They were named according to the sampling site (“ARG” for Brest Bay, “LTB” for La Tremblade in Marennes-Oleron Bay, “ARC” for Arcachon Bay and “THAU” for Thau Lagoon) followed by the number of the isolate. The 16 S rRNA gene sequence was obtained for 293 strains. The identified bacteria were divided into the following phyla: *Proteobacteria* (62.8%), *Firmicutes* (15.3%), *Bacteroidetes* (12.3%) and *Actinobacteria* (9.6%) (Fig. 2). The three major genera were *Vibrio*, *Bacillus* and *Shewanella* (Fig. 2). The majority of the isolated species were found at all sites.

In parallel, in silico correlation analysis was performed to predict which bacteria were preferentially associated with resistant or sensitive oysters. This LefSE analysis^60^ was performed based on previously published 16 S rRNA gene barcoding datasets which describe the bacterial part of the bacterial microbiota community isolated from 687 POMS-resistant and 664 POMS-sensitive oysters (**Supplementary File 1**,** Table ****S3**). Based on this analysis, 118 bacterial genera were shown to be preferentially associated with POMS-resistant oysters (**Supplementary File 1**,** Table ****S5**). By combining the data obtained from this predictive in silico analysis and data from the scientific literature about bacteria shown to be beneficial in an aquaculture context^73,74,83,84,75–82^, we selected 12, 17, 10 and 8 bacteria for the Brest, La Tremblade, Arcachon and Thau sites respectively (Table 1). These bacterial strains were then tested for their cytotoxic effects on 2 day-old larvae. The most cytotoxic bacteria were discarded. Based on these results, five, seven, five and five site-specific bacteria were used to produce the Brest, La Tremblade, Arcachon and Thau multi-strain bacterial mixes respectively (Table 1). A fifth multi-site bacterial mix was produced from bacteria isolated from oysters sampled at all sites. For this purpose, seven different bacteria were chosen because they displayed the least cytotoxic effects on larvae (Table 1).

In summary, we collected bacteria from disease-resistant oysters. We then combined our findings with existing literature and utilised in silico predictive analysis. This allowed us to create four site-specific and one multi-site multi-strain bacterial mixes, all of which have the potential to benefit oyster health.

**Table 1:** Composition of the 5 multi-strain bacterial mixes produced according to their predictive beneficial properties.

Environment	Collection of bacteria		Nb. of genera selected for cytotoxic assay on larvae	Nb. of bacteria selected after cytotoxical assay	Multi-strain bacterial mixes	
	Nb. of bacteria in the collection	Nb. of genera			Names	Strains
Brest	97	40	12	5	Brest Mix	*Shewanella sp.* ARG21
						*Marinibacterium sp.* ARG39
						*Shewanella sp.* ARG89
						*Shewanella sp.* ARG96
						*Shewanella sp.* ARG129
La Tremblade	144	45	17	8	La Tremblade Mix	*Halomonas sp.* LTB66
						*Neptunomonas sp.* LTB74
						*Psychrobacter sp.* LTB83
						*Paracoccus sp.* LTB95
						*Halomonas sp.* LTB102
						*Cobetia sp.* LTB109
						*Sulfitobacter sp.* LTB127
Arcachon	56	26	10	5	Arcachon Mix	*Shewanella sp.* ARC21
						*Bacillus sp.* ARC34
						*Colwellia sp.* ARC55
						*Neptunomonas sp.* ARC59
						*Tenacibaculum sp.* ARC64
Thau	67	18	8	5	Thau Mix	*Shewanella sp.* THAU5
						*Paracoccus sp.* THAU19
						*Ruegeria sp.* THAU28
						*Shewanella sp.* THAU34
						*Paracoccus sp.* THAU46
					Multi-site Mix	*Marinibacterium sp.* ARG39
						*Shewanella sp.* ARG89
						*Halomonas sp.* LTB57
						*Cobetia sp.* LTB109
						*Neptunomonas sp.* ARC59
						*Paracoccus sp.* THAU19
						*Paracoccus sp.* THAU46

### The microorganism exposures during larval rearing stages displayed from moderate to strong effects on larval survival

The multi-strain bacterial mixes were added to four oyster populations during the larval rearing. The four populations were the sympatric oysters from which the bacteria were isolated (i.e., Brest, La Tremblade, Arcachon and Thau). An exposure with a whole microbiota community coming from healthy hatchery donor oysters was also performed (ME seawater D0-D14). These oysters were shown to be devoid of the three main pathogens (*V. coralliilyticus*, OsHV-1 µVar and *Haplosporidium costale*) of *C. gigas* from larvae to juveniles^10,44^. Oyster larvae were exposed to bacterial mixes either from the blastula stage (3 h post-fertilisation (pf)) to pediveliger stage (14 days pf) (D0 to D14) or from veliger stage (seven days pf) to pediveliger stage (14 days pf) (D7 to D14) (Fig. 1). Moderate mortalities during the larval rearing was observed when the larvae were exposed to Thau D0-D14, Brest D7-D14, La Tremblade D7-D14 and the Multi-site D7-D14 mixes, with mean survival rates of 90.3%, 98.9, 70.5 and 73% respectively compared to the control). Intermediate mortality was observed when the larvae were exposed to Arcachon D0-D14 and ME D0-D14 with mean survival rates of 49% and 38.8% respectively. Severe mortality was observed when larvae were exposed to Brest D0-D14, La Tremblade D0-D14 and multi-site D0-D14 mixes leading to the total loss of Thau oyster exposed to these 3 bacterial mixes.

**Fig. 3:**
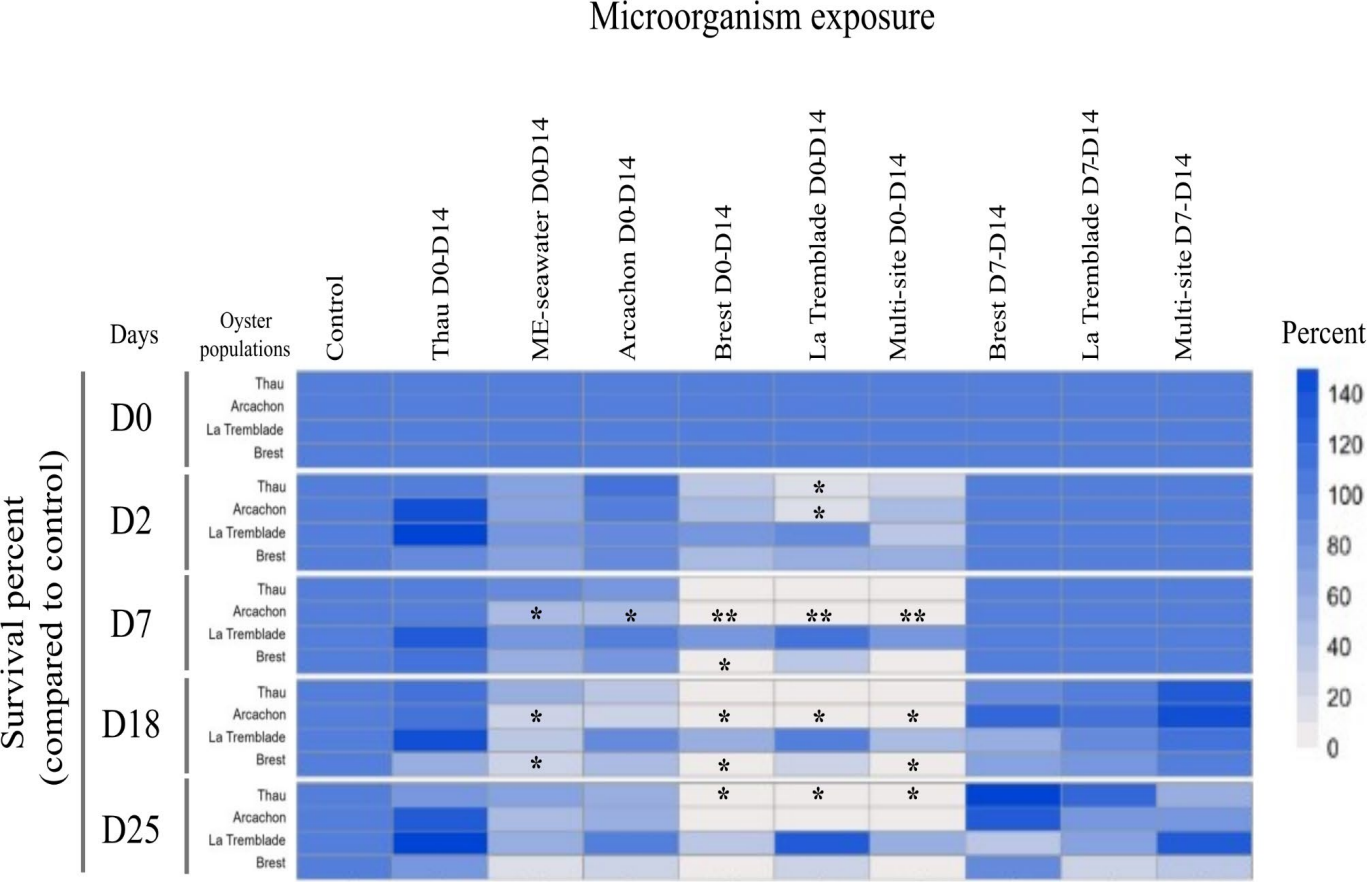
Multi-strain bacterial mixes displayed contrasted effects on larval survival. The effects of the exposure on the survival of larvae from the four oyster populations at different times of the larval rearing stages were recorded. The heatmap shows the survival (in percent) of the larvae exposed to microorganisms compared to the control condition. The asterisks (*) inside the box indicate statistical differences compared to control condition (* p < 0.05; ** p < 0.01; *** p < 0.0﻿01﻿).

Overall, these microorganism exposures during larval rearing stages displayed from moderate to severe effects on larval survival. These effects rely on oyster origin and, also, on the bacterial content of the microorganism exposure. In addition, the window of exposure, from day 7 to day 14, induced less mortality compared to an earlier window starting 3 h after fertilisation (D0-D14) (Fig. 3**and Supplementary File 5 Effect of bacterial mixes on oyster larvae)**.

.

Subsequently, each oyster population (exposed and control) were challenged with OsHV-1 µVar infection during the juvenile stage or *V. aestuarianus* during the adult stage. The success of the experimental infection was verified by quantifying the viral or Vibrio DNA concentration in the sea water of the experimental tanks (**Supplementary File 1**,** Table ****S6**** and Table S7**). The Thau oysters exposed to the Brest mix D7-D14 and ME-seawater D0-D14 were not challenged due to technical problems which arose in the nursery.

**Fig. 4:**
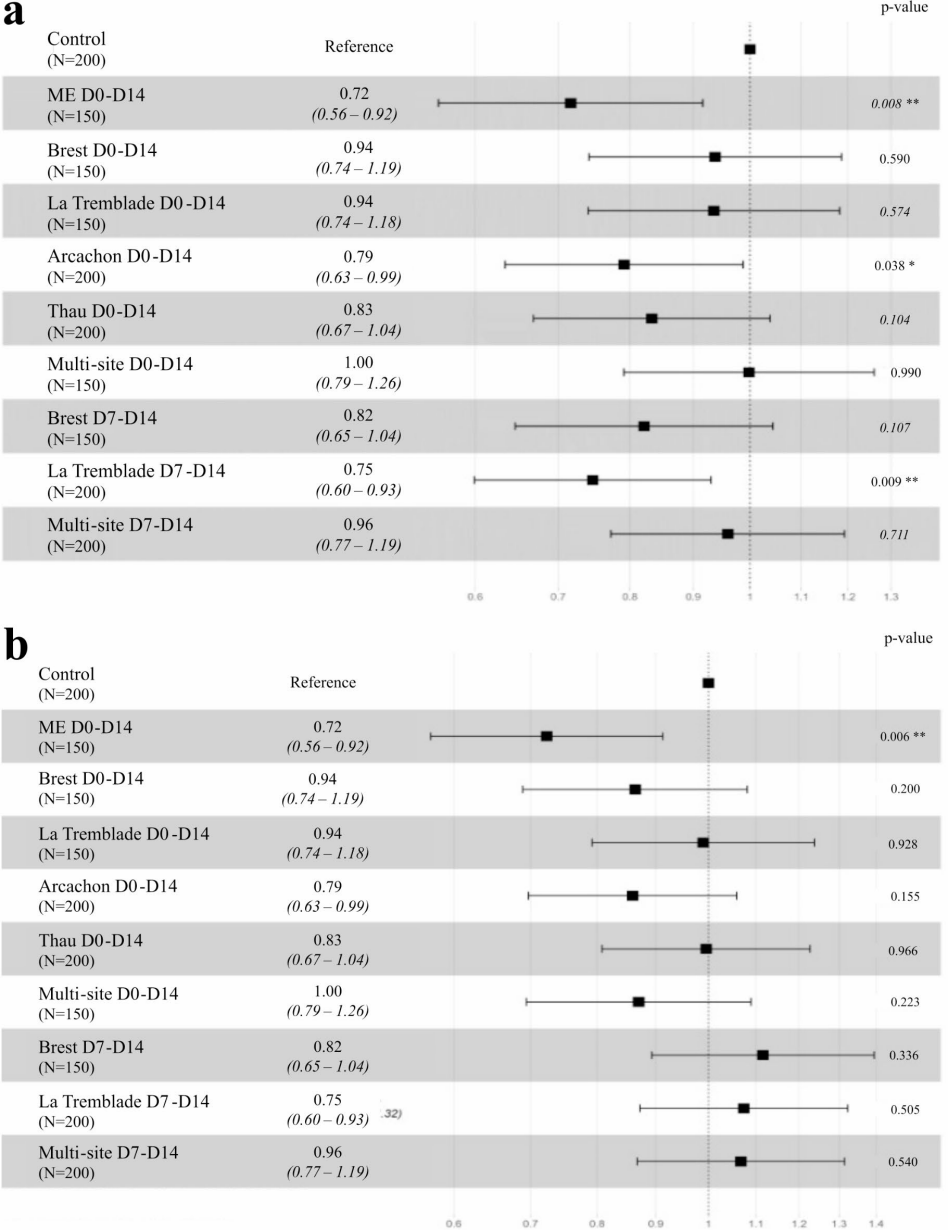
Bacterial mixes and ME-seawater exposure during larval rearing reduce the mortality risk induced by POMS or***V. aestuarianus***. Forest plot ﻿representing the Hazard-Ratio value of mortality risk during the (**a**) OsHV-1 µVar and (**b**) *V. aestuarianus* experimental infection for oysters (all populations combined) exposed to microorganisms compared to control oysters. The numbers in brackets under the different conditions correspond to the number of oysters used during the experimental infection. The Hazard-Ratio value is indicated to the right of the conditions, except for the control condition, which is indicated as a reference. The p-value of the log rank test is indicated on the rig﻿ht-hand side of each row.﻿

In response to OsHV-1 µVar infection, a significant reduction in mortality risk of 21% (Log-rank test: pval = 0.038), 25% (Log-rank test: pval = 0.009), and 28% (Log-rank test: pval = 0.008) was observed in the oysters (all populations combined) exposed to the Arcachon D0-D14, La Tremblade D7-D14 and D0-D14 ME seawater mixes, respectively (Fig. 4a). We observed that the mortality started 3 days after the POMS disease induction, and differences between the control and exposed samples appeared as soon as mortality started for oysters exposed to the Arcachon D0-D14, La Tremblade D7-D14 and, ME seawater D0-D14 oysters (**Supplementary File 3**,** Figure ****S2**).

In response to vibriosis, a significant reduction in the mortality risk of 28% (Log-rank test: pval = 0.006) was observed for the ME seawater D0-D14 exposed oysters (Fig. 4b) (**Supplementary File 3**,** Figure ****S3**). Other exposures did not lead to reduction of mortality.

For both vibriosis and viral infection, the beneficial effect in response to each of the mixes depended on the oyster origin (**Supplementary File 3**,** Figure ****S2****)**. Oysters originating from Arcachon showed the greatest reduction in mortality in response to both infections regardless of the bacterial exposure conditions during the larval stages. The effect of microorganism exposure was intermediate on oysters from La Tremblade and less pronounced on the oysters from Brest and Thau (**Supplementary File 3**,** Figure ****S2**).

In summary, protection against POMS disease was achieved when the larvae were stimulated either with bacterial mixes or with Microorganism-Enriched seawater (ME seawater), while protection against Vibriosis was obtained only with stimulation by Microorganism-Enriched seawater. No preferential beneficial effect was nevertheless observed when the oysters were exposed to their sympatric compared to allopatric strains.

### Microorganism exposure during larval rearing induced long term changes of the microbiota composition

To test the immediate and long-term effects of microorganism exposure on the oyster microbiota composition, we analysed the bacterial communities by 16 S rRNA gene sequencing during the larval stage after seven days of exposure and during the juvenile stage seven months after the exposure. We focused our study on the three conditions of bacterial exposure that conferred significant increase on the survival of oysters during OsHV-1 µVar and *V. aestuarianus* experimental infection.

Sequencing of the V3-V4 hypervariable region of the 16 S rRNA gene resulted in a total of 10,868,202 clusters. After a quality check (deleting primers and low-quality sequences, merging, and removing chimeras) and ASV clustering, 5,322,399 reads (49%) with an average of 35,962 reads per sample were retained for downstream analyses.

A greater species richness was observed seven days after the exposure for ME-seawater exposed larvae but not after exposure to the bacterial mixes (**Supplementary File 3**,** Figure ****S4**** a**,** b**). This difference was not maintained at the juvenile stage **(Supplementary File 3**,** Figure ****S4**** c)**. Dissimilarity analysis, based on the Bray-Curtis index, showed that the larvae microbiota composition differed between conditions after seven days of microorganism exposure, regardless of the condition (Table 2**)**. This difference remained statistically significant at the juvenile stage for ME seawater D0-D14 and La Tremblade D7-D14 conditions (Table 2**)**.

We additionally checked for the presence of the added bacteria during the larval stage, after seven days of exposure, with the last addition of bacteria performed 48 h before sampling, and at the juvenile stage seven months post-exposure. Two bacterial strains out of the 5 added to larvae exposed to Arcachon D0-D14 were retrieved and represented 3.3 to 25.9% of the total bacterial community (**Supplementary File 3**,** Figure ****S5**** a**). ASVs associated with the added bacteria of the La Tremblade D7-D14 ranged from 0.09 to 0.96% in the corresponding larvae samples (**Supplementary File 3**,** Figure ****S5**** c**). None of the ASVs corresponding to bacteria used for the exposure could be detected at the juvenile stages seven months post-exposure (**Supplementary File 3**,** Figure ****S5**** b**,** d)**. Furthermore, either for larvae or juvenile oysters, bacterial strains did not show a preference for implantation in their sympatric host population (**Supplementary File 3**,** Figure ****S5**). Using this pipeline of detection, we were able to detect these ASVs on a mock control containing an artificial mix of bacteria in the same proportion except for *Paracococcus* sp. LTB95 and *Psychrobacter* sp. LTB83 (**Supplementary File 3**,** Figure ****S6****)**. This indicated that the lack of detection of the ASVs in exposed oyster is due to an absence of the bacteria rather than a technical shortcoming in our detection pipeline, except for *Paracococcus* sp. LTB95 and *Psychrobacter* sp. LTB83.

In summary, a few proportions of the different bacteria that were added during the larval rearing were detected in the oyster microbiota 48 h after the last addition of bacteria, and none of them were maintained on a long-term basis. Despite this lack of bacterial colonisation, the overall composition of the microbiota was modified in response to bacterial exposure and these changes persisted until the juvenile stage.

**Table 2:** Long-lasting modifications in *C. gigas* microbiota composition occurred following microorganism exposure. Permanova (adonis2) on the Bray-Curtiss dissimilarity matrix showing the effects of microbial exposure on microbiota community compared to the control condition for larvae after seven days of microbial exposure and for juveniles seven months after microbial exposure. For larvae, analyses were performed on all oyster populations confounded which represent eight pools of 10,000–20,000 larvae sampled in eight independent tanks for exposure to ME D0-D14 and Arcachon D0-D14 and on four pools of 10,000–20,000 larvae sampled in four independent tanks for exposure to La Tremblade D7-D14. For the juvenile stages, analyses were performed on all oyster population confounded which represent 68 individuals sampled in five independent tanks.

Conditions (Compared to control)	Larvae (after 7 days of exposure)				Juvenile (7 months)			
	Dum Sq	R²	F	*p*	Dum Sq	R²	F	*p*
ME D0-D14	0.75	0.18	4.46	0.001	0.19	0.04	1.45	0.026
Arcachon D0-D14	0.85	0.19	3.30	0.001	0.08	0.02	0.75	0.908
La Tremblade D7-D14	0.43	0.29	2.98	0.036	0.17	0.04	1.55	0.033

### Microorganism exposure during larval rearing induced long-term changes in oyster immunity

The long-term impact of microorganism exposure on oyster gene expression was analysed by RNA-seq on juvenile oysters before and during POMS challenge. In total, RNA sequencing produces between 15.1 and 36.6 million reads per sample (mean number of reads = 26 million). Among these reads, 67.28–77.52% were mapped to the *C. gigas* reference genome (assembly cgigas_uk_roslin_v1) (**Supplementary File 4: RNAseq Mapping result)**.

**Fig. 5:**
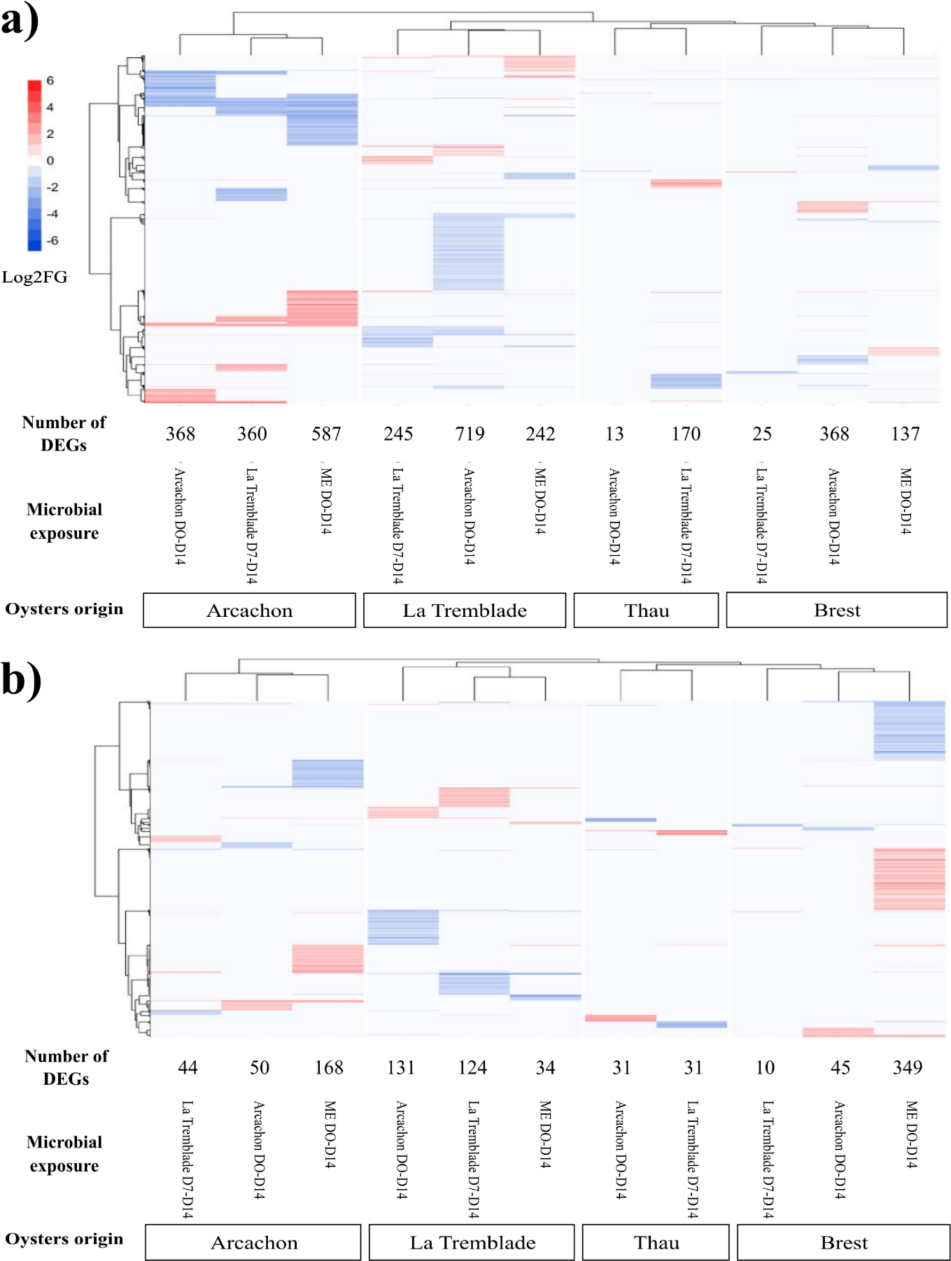
Specific gene expression profiles were observed in response to each microorganism exposure. Heatmap of differentially expressed genes (DEGs) in oysters exposed to Arcachon D0-D14, La Tremblade D7-D14 or ME seawater D0-D14 compared to control oysters for the four oyster populations (Brest, Arcachon, La Tremblade and Thau) (**a**) prior to OsHV-1 µVar infection and (**b**) 3 h post-OsHV-1 µVar infection. The intensity of DEG ratios is represented by the Log2 Fold Changes (Log2FC) for over expressed DEGs (in red) and under expressed DEGs (in blue). n = 3 individuals per﻿ condition.

For each of the four oyster populations, the number of differentially expressed genes (DEGs) in oysters exposed to bacterial mixes or to ME seawater compared to control oysters, was higher before the infection than 3 h after the beginning of the infection except for the condition where Brest oysters were exposed to ME seawater (Fig. 5). Furthermore, each oyster population displayed a specific transcriptomic response, which strongly varied according to microorganism exposure. (Fig. 5).

**Fig. 6:**
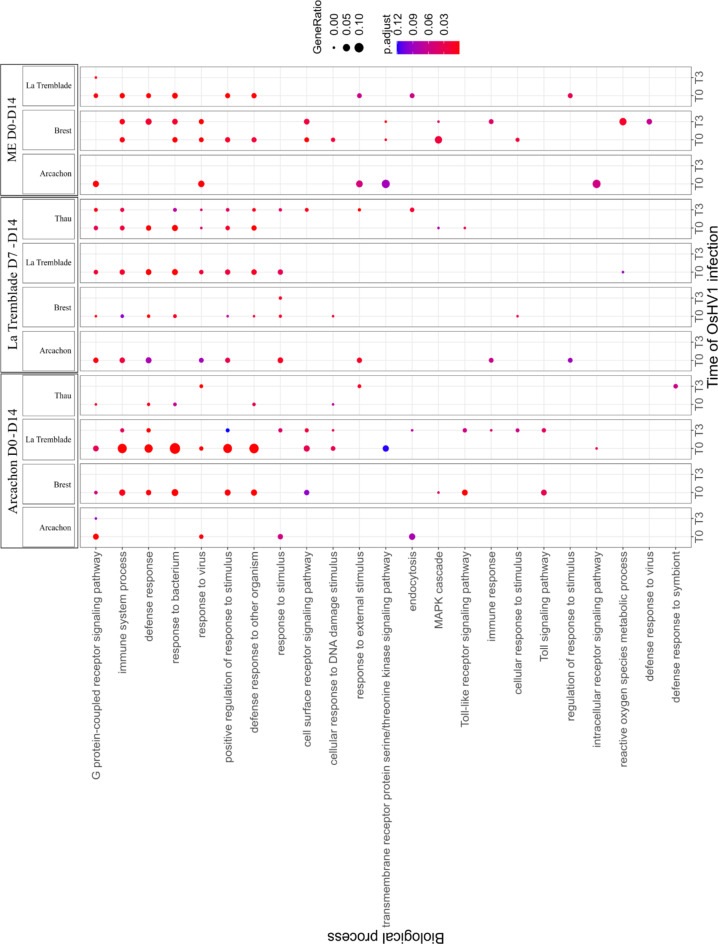
GO term enrichment analysis revealed important immune pathways modified in response to the microorganism exposure. Dot plot showing the overrepresented GO terms (FDR < 0.1) of biological process (BP) related to immune function identified using GO_MWU for the four oyster population (Brest, Arcachon, La Tremblade and Thau) exposed to Arcachon D0-D14, La Tremblade D7-D14 or ME seawater D0-D14 compared to control oysters at t = 0 and t = 3h of OsHV-1 µVar infection. The dot size is proportional to the number of differentially expressed genes (DEG) in the biological process compared to the control condition, and the colour of the dot indicates the significance.

**Fig. 7:**
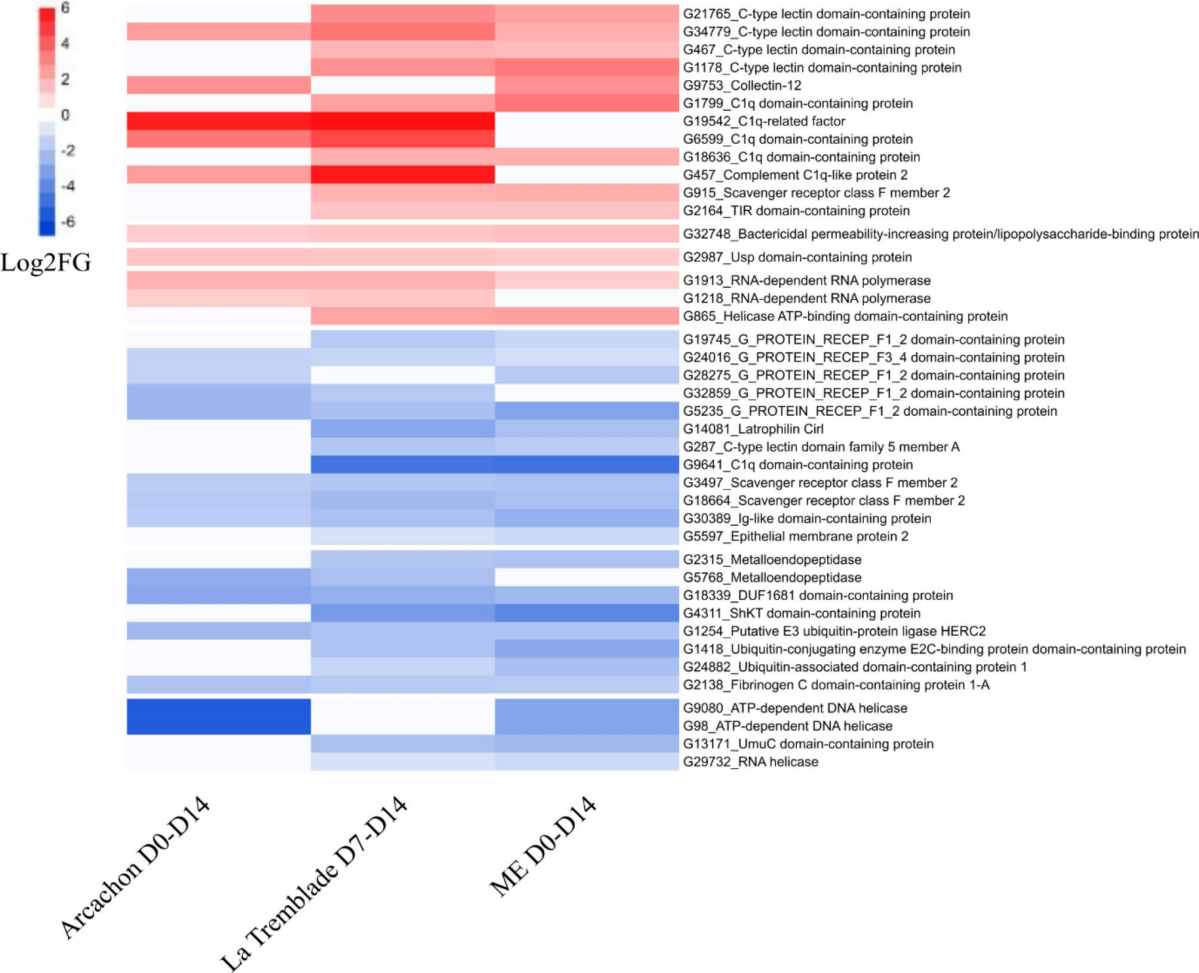
Detailed immune-related gene expression revealed key genes modified in Arcachon oysters in response to microorganism exposure. Transcriptomic response of immune related genes for oysters of the Arcachon population exposed to Arcachon D0-D14, La Tremblade D7-D14 or ME seawater D0-D14 compared to control condition before OsHV-1 µVar experimental infection. Heatmap of DEGs associated with immune processes. Only DEGs found under at least two conditions of exposure to micro-organisms were shown. The intensity of DEG ratios is expressed in Log2 Fold changes (Log2FC) for over expressed DEGs (in red) and under expressed DEGs (in blue).

To identify which biological processes were affected by microbial exposure, we conducted a Rang-Based Gene Ontology Analysis (GO_MWU)^71^. The range of biological process enriched in DEGs (microorganisms exposed vs. control) before and during the onset of POMS disease included many GO terms such as, metabolism, RNA and DNA process, protein processing, signal transduction, transport, and immune functions. We then focused on the enriched immune functions in oysters exposed to microorganisms compared to the control oysters (Fig. 6). The most significantly enriched functions related to immunity across all oyster populations and all treatments were general functions of immunity (defence response, immune system process), functions related to the response to organisms (response to bacterium, response to virus), a function related to the positive regulation of response to stimulus and a function related to the G-protein signalling pathway (Fig. 6). As the oysters from Arcachon showed the greatest reduction in mortality risk in the face of viral infection and *V. aestuarianus*, with all the microbial exposures, we then analysed, for these oysters only, the individual DEGs for the main enriched functions linked to immunity described in (Fig. 6). This analysis revealed that before the infection (t = 0), gene coding for Pattern Recognition Receptor (PRRs) (C-type lectins, C1q domain containing protein), innate immune pathways (toll-interleukin receptor (TIR), Complement pathway), interaction with bacteria (Bactericidal permeability-increasing protein) and antiviral pathways (RNA and DNA Helicases, RNA-dependent RNA polymerase) were found to be over-represented in microbial exposed oysters compared to control oysters (Fig. 7) (**Supplementary File 6 List of DEGs**).

In summary, long-lasting changes in gene expression were observed in juvenile oysters seven months after they had been exposed to bacterial mixes or Microorganisms Enriched seawater during the larval stage. The long-lasting transcriptional responsiveness was found to be influenced by the host’s origin, was specific to the type of treatment and significantly impacts the host immune response.

## Discussion

OsHV-1 µVar, a pathogen that threatens oyster production, has spread not only to Europe^6,7^ but also to the United States^4^, Japan^85^, Australia^86^, China^87^ and New-Zealand^21^. On the other hand, the pathogenic bacterium *V. aestuarianus* has been observed to spread across Europe^88^ and in North America (British Columbia, Canada)^89^. Innovative research and concerted efforts are currently being explored for safeguarding *C. gigas* and ensuring the sustainability of oyster farming on a global scale^12,13,15,18^. One promising avenue of research involves education of the oyster immune system through proper setting of the microbiota during early life. Similar to the way early microbial colonisation impacts human health^30,32^, introducing specific microorganisms to oyster larvae can potentially educate their innate immune system and improve disease resistance^20,37^. The immune system in oysters is set up early during the development since the existence of a primitive immune system has been detected in the trochophore larvae^90,91^. This microbial education plan is a promising strategy as it is easy to implement, not costly and, can be performed on numerous animals (several hundred million of larvae) at the same time by bath or on their diet. However, a challenge arises in the form of current hatchery practices, which aim to minimise the introduction of both non-pathogenic and pathogenic microorganisms into larval tanks^92–95^. Mortality issues, particularly during larval rearing, have led to the use of antibiotics in hatcheries. Therefore, finding a balance between educating the immune system and addressing concerns about uncontrolled microbiota transfer is crucial. Here, our study explored the feasibility of microbial education in oyster larvae while considering and mitigating the risks associated with the uncontrolled transfer of hazardous microorganisms.

For this purpose, we investigated the long-term protection conferred by a larval exposure to a controlled non-pathogenic whole microbiota transferred from donor oysters. The donor oysters used in this study were always kept in biosecured facilities. In this way, the oysters were shown to be devoid of the three main pathogens of *C. gigas* from larvae to juveniles^10,44^. In parallel, we performed the same assay using a reduced, synthetic bacterial community composed of cultivable bacteria. Cultivable bacteria were isolated from POMS-resistant oysters and selected according to their predictive beneficial effect on POMS disease based on robust correlation analysis. The microbial exposure was performed on 4 different oyster populations, each exposed to either a sympatric or allopatric multi-strain bacterial mixes. We showed that larval exposure to a whole microbiota from donor oysters provided protection against both POMS disease and *V. aestuarianus* infection. In a different way, larvae exposed to multi-strains bacterial mixes showed improved survival against OsHV-1 µVar but no protection against *V. aestuarianus* infection. This can be explained by the fact that our bacteria were selected from oysters resistant to POMS disease, probably leading to a bias in favour of protecting against POMS disease. The host origin was identified as a critical factor for the protection conferred and no preferential effect was observed when sympatric multi-strains mixes were used. This work demonstrates the potential of leveraging the oyster microbiota to enhance long-term disease resistance in oysters and sheds light on the importance of considering the host origin in such protective mechanisms.

Targeting early developmental stages as a strategic window for probiotic application to induce long-term protection has been proposed and explored in various animal models, such as mammals or humans (see review by^96^), as well as those relevant to livestock production^97,98^. Introducing beneficial microorganisms during these stages can influence both the host’s microbiota composition and immune system development, potentially leading to long-term beneficial immunomodulation. Our results are in line with these findings since we observed a shift in both the transcriptional pattern and microbiota composition of oysters exposed to beneficial microorganisms compared to their non-exposed counterparts, even seven months after the exposure. Nevertheless, although our results do not allow us to define the optimal exposure window conferring the best protective effect, we have demonstrated the importance of carefully studying and determining this most suitable window and the duration of microbial exposure in order to avoid detrimental effects during the larval rearing. Our findings indicate that the earliest window (starting 3 h after fertilisation) is particularly sensible to the microorganism exposure. Depending on the bacterial content and the oyster’s genetic background, this exposure might lead to severe mortality. The long-lasting transcriptional responsiveness was found to be influenced by the host’s origin and was specific to the type of microbial treatment administered. A significant portion of the differentially expressed genes in exposed oysters were associated with immune functions, with a particular emphasis on pattern recognition receptors (PRRs). Intriguingly, the observed difference in phenotype between oysters stimulated with the whole microbiota and those stimulated with multi-strain bacterial mixes could not be fully explained through a thorough analysis of the differentially expressed genes. This suggests that additional factors or intricate interactions within the oyster’s immune system and microbiota may contribute to the differential response. Furthermore, when the oysters were challenged with OsHV-1 µVar three hours after exposure, changes in the transcriptional pattern were still evident in oysters exposed to beneficial microorganisms compared to their non-exposed counterparts, albeit to a lesser extent than before the infectious challenge. This indicates a dynamic interplay between the immune response against the virus and the prior microbial stimulation, with the virus potentially exerting a more pronounced effect on the transcriptional response.

Our findings further indicate that exposure to either bacterial mixes or whole microbiota leads to changes in the microbiota composition. This was observed during the exposure but also on a long-term basis as previously observed in other studies^23,97,99^. Interestingly, the bacteria added as part of the bacterial mixes were not detected using the employed method. This suggests that the added bacteria did not effectively integrate the oyster microbiota, even shortly after the start of the exposure. Similar studies indicate that administered bacteria fail to establish and only persist temporarily in the microbiota of exposed animals. For instance, the *Aeromonas* sp. strain administered to oyster larvae was undetectable 72 h after addition^100^. Similarly, exposing the European abalone (*Haliotis tuberculata*) to the *Pseudoalteromonas* sp. hCg-6 exogenous strain resulted in a transient establishment of the probiotic strain in the haemolymph rather than a sustained interaction^101^. Additionally, Arctic Char (*Salvelinus alpinus*) exposed to various probiotic strains did not show detectable levels of the administered strains four weeks after probiotics administration^102^. The change in microbiota composition observed on long-term basis might thus be linked to ongoing interactions between the microbiota and the immune system, leading to a continuous reshaping of both elements and explaining also the observed long-term transcriptional changes.

## Conclusion

Our study successfully investigated methods which aimed at exposing oysters to specific beneficial microorganisms during larval rearing to educate their immune system. We considered the potential risks associated with microbial exposure while ensuring that the oysters’ innate immune system was primed for improved disease resistance. We demonstrated the potential of leveraging this microbial education to enhance disease resistance to two major oyster pathogens, OsHV-1 µVar and *V. aestuarianus*, which are currently critical threats for oyster farming worldwide. Additionally, our findings emphasise the potential of using controlled whole microbiota transfers as the best strategy to safeguard oyster health in aquaculture settings.

Additional optimisations will be required to identify the most effective settings for enhancing the beneficial impact of microbial education. The timing, duration of exposure, and rearing conditions are essential factors for the practical application of this approach in aquaculture environments. Exploring combinations with other strategies, such as selecting oysters with genetic backgrounds that are more receptive to microbial education, is another avenue that certainly deserves further investigation.

## Electronic supplementary material

Below is the link to the electronic supplementary material.

Supplementary Material 1

Supplementary Material 2

Supplementary Material 3

Supplementary Material 4

Supplementary Material 5

Supplementary Material 6

## Data Availability

Raw sequence data for RNA-seq and 16S sequencing for metabarcoding analysis have been made available through the SRA database (BioProject accession number PRJNA1078733).

## Abbreviations

ASV: Amplicon Sequence Variants
CFU: Colony‑forming unit
DEG: Differentially expressed gene
HR: Hazard-Ratio
LEfSe: Linear discriminant analysis (LDA) Effect Size
MB: Marine Broth
NSI: Naissains Standardisés Ifremer or standardised Ifremer spats
OsHV-1 µVar: Ostreid Herpes Virus 1 µVar
OTU: Operational Taxonomic Unit
pf: post-fertilisation
POMS: Pacific Oyster Mortality Syndrome
RNA‑Seq: Sequencing of the polyadenylated ribonucleic acids
